# In sickness and in health: the role of methyl-CpG binding protein 2 in the central nervous system

**DOI:** 10.1111/j.1460-9568.2011.07658.x

**Published:** 2011-05

**Authors:** Sol Díaz de León-Guerrero, Gustavo Pedraza-Alva, Leonor Pérez-Martínez

**Affiliations:** Laboratorio de Neuroinmunobiología, Departamento de Medicina Molecular y Bioprocesos, Instituto de Biotecnología, Universidad Nacional Autónoma de MéxicoA.P. 510-3, Cuernavaca, Morelos 62271, México

**Keywords:** chromatin remodeling, epigenetics, mental retardation, microRNAs, Rett syndrome, signal transduction neurodevelopmental disorders

## Abstract

The array of specialized neuronal and glial cell types that characterize the adult central nervous system originates from neuroepithelial proliferating precursor cells. The transition from proliferating neuroepithelial precursor cells to neuronal lineages is accompanied by rapid global changes in gene expression in coordination with epigenetic modifications at the level of the chromatin structure. A number of genetic studies have begun to reveal how epigenetic deregulation results in neurodevelopmental disorders such as mental retardation, autism, Rubinstein–Taybi syndrome and Rett syndrome. In this review we focus on the role of the methyl-CpG binding protein 2 (MeCP2) during development of the central nervous system and its involvement in Rett syndrome. First, we present recent findings that indicate a previously unconsidered role of glial cells in the development of Rett syndrome. Next, we discuss evidence of how MeCP2 deficiency or loss of function results in aberrant gene expression leading to Rett syndrome. We also discuss MeCP2's function as a repressor and activator of gene expression and the role of its different target genes, including microRNAs, during neuronal development. Finally, we address different signaling pathways that regulate MeCP2 expression at both the post-transcriptional and post-translational level, and discuss how mutations in MeCP2 may result in lack of responsiveness to environmental signals.

## Introduction

Understanding the molecular mechanisms that control the phenotypic identity of distinct neuronal classes at defined regions within the central nervous system constitutes a widely relevant issue in developmental neuroscience. Neurons permanently exit the cell cycle and remain quiescent. Neuronal mitotic quiescence is crucial for the maintenance of the complexly wired brain. Therefore, neuronal differentiation encompasses an elaborate developmental program in which neurogenic and antiproliferative signals work in concert to ensure the differentiated state (Perez-Martinez & Jaworski, 2005).

**Fig. 1:**
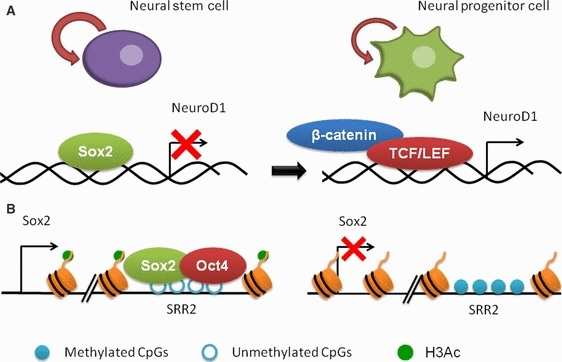
Neuronal differentiation results from specific gene expression regulation. (A) In neural stem cells (NSCs), sex determining region Y-box 2 (Sox2) is highly expressed and represses the transcription of neuronal genes like Neurogenic differentiation 1 (NeuroD1). As cells differentiate from NSCs to neural progenitor cells, Sox2 expression is lost and NeuroD1 is activated by wingless-type MMTV integration site family member (Wnt) signaling-dependent binding of T-cell factor/lymphoid enhancer factor (TCF/LEF) to its promoter (Kuwabara *et al.*, 2009). (B) In proliferating neural cells, Sox2 and Oct4 (also known as POU5F1 for POU class 5 homeobox 1) are highly expressed and together regulate a wide variety of genes, including Sox2 itself. Sox2 and Oct4 bind to an enhancer region (named SRR2) located downstream of Sox2 gene, which is found in an unmethylated state. Sox2 and Oct4 occupancy also correlate with open chromatin marks (H3Ac) in the enhancer region and throughout the gene. As cells differentiate into neurons, Sox2 and Oct4 occupancy on the SRR2 enhancer is lost, the CpGs in the enhancer become methylated and the open chromatin marks (H3Ac) are lost (Chew *et al.*, 2005).

During development, mitotically active precursors located within the neuroepithelium give rise to all the neuronal and glial cells of the brain. Recent genetic studies in vertebrate and invertebrate models have shown that neuronal differentiation is under the control of complex regulatory networks of transcription factors, which are activated in response to inductive signals. The coordinated actions of these proteins direct neuronal fate identity in appropriate spatial and temporal contexts during central nervous system development (Jurata *et al.*, 2000; Lee & Pfaff, 2001; Fig. 1).

Environmental cues, via specific intracellular signaling pathways, control neuronal differentiation at multiple levels: neuronal precursor cell cycle control, migration, synaptogenesis, neuronal survival and neurotransmitter phenotype. As mentioned above, these cellular responses result from the induction of timely coordinated cascades of gene expression controlled by specific and ubiquitous transcription factors. These transcription factors in turn induce either the expression of structural genes required for specific functions of a given neuronal lineage and/or the expression of non-coding RNAs that play an important regulatory role. Among the small non-coding RNAs identified so far are those with (i) gene-silencing effects [small interfering RNAs (siRNAs) and microRNAs (miRNAs)] at the post-transcriptional level and (ii) small modulatory double-strand RNAs (smRNAs) that regulate gene expression at the transcriptional level through double-stranded RNA/protein interactions (Mattick & Makunin, 2005). In mammals, certain miRNAs have been implicated as important regulators in the maintenance of the pluripotent cell state during early development (Houbaviy *et al.*, 2003) and neurogenesis (Sempere *et al.*, 2004). However, recent studies have demonstrated that the action of transcription factors mediating neuronal differentiation depends on specific epigenetic traits like histone post-translational modifications, polyADP-ribosylation and DNA methylation. Here, we review the latest studies aimed at understanding how an epigenetic deregulation results in neurodevelopmental disorders. Specifically, we focus on the role of the methyl-CpG binding protein 2 (MeCP2) in neuronal development and disease. We also discuss different MeCP2 targets including miRNAs, and mechanisms of MeCP2 regulation.

## Methylation and gene expression

Developmental stages in mammals are established by genetic and epigenetic programs. Epigenetic information is normally defined as those processes that are inherited through mitosis or meiosis influencing gene expression independently of the DNA sequence. They constitute signals that are interpreted to regulate gene expression and cellular differentiation, among other cellular processes. The chromatin structure provides an additional level of regulation over gene expression as chromatin can switch between a closed or transcriptionally repressive state, to an open or transcriptionally permissive state (Berger, 2007). These changes in chromatin architecture are regulated by different processes including chromatin remodelers (Clapier & Cairns, 2009), histone variants (Kamakaka & Biggins, 2005), histone post-translational modifications (Kouzarides, 2007) and DNA methylation (Miranda & Jones, 2007). DNA methylation is a covalent modification that, in the case of vertebrates, consists of the addition of a methyl group to the 5′ carbon of cytosine residues (Turek-Plewa & Jagodzinski, 2005). Cytosine methylation normally occurs in the context of the palindromic 5′-CG-3′ (CpG) dinucleotide (Holliday & Pugh, 1975; Bird, 1978). In mammals, between 60 and 90% of all CpGs are methylated (Bird, 1986). In vertebrates, there are CpG-rich regions referred to as CpG islands, which are predominantly located in regulatory regions. These islands can be found in approximately 60% of all mammalian genes (Antequera & Bird, 1993), and are mostly found in an unmethylated state (Bird, 1986). DNA methylation patterns vary significantly throughout development. Cytosine methylation is established and maintained by DNA methyltransferases (Dnmts), whose expression is tightly regulated during development (reviewed in Goll & Bestor, 2005). In mammals, *de-novo* methylation is carried by two enzyme families: Dnmt3a and Dnmt3b (Okano *et al.*, 1999). Both proteins are essential for normal development as *Dnmt3a* knock-out mice become runted after birth and die at around 4 weeks of age; *Dnmt3b* knock-out shows embryonic defects and causes death before embryonic day 15.5. Homozygous animals, mutant for both *Dnmt3a* and *Dnmt3b*, exhibit a more severe phenotype than individual mutants; they die before embryonic day 11.5, suggesting an overlapping function for these proteins in embryogenesis (Okano *et al.*, 1999).

Following replication, when the newly synthesized DNA strand is unmethylated, an enzyme binds to hemimethylated sites and catalyzes the transfer of a methyl group on the daughter strand to restore the palindromic methyl CpG configuration (Leonhardt *et al.*, 1992). This maintenance methylation activity is catalyzed by Dnmt1 and ensures that the established methylation patterns are preserved over many cell generations, and provides a means for heritable transcriptional control during development. Dnmt1 is expressed throughout embryonic development; in the adult stage Dnmt1 exists as two isoforms, Dnmt1 and Dnmt1b (Bonfils *et al.*, 2000).

DNA methylation has been shown to play an important role in several processes, including the suppression of tissue-specific genes and imprinted genes, X chromosome inactivation, and stability of transposable elements (Jaenisch *et al.*, 1985; Keshet *et al.*, 1986; Becker *et al.*, 1987; Li *et al.*, 1993; Panning & Jaenisch, 1996; Walsh *et al.*, 1998). It has also been shown that methylation confers additional stability to the genome by providing a more tightly packed chromatin, in particular along repetitive sequences and constitutive heterochromatic regions to avoid unspecific recombination events (Colot *et al.*, 1996; Chen *et al.*, 1998; Hashimshony *et al.*, 2003). The most common effect of DNA methylation is gene silencing, which can be achieved in two ways: (i) a transcription factor binding site can be altered by the presence of a methylated cytosine, preventing its recognition and binding by the transcription factor; and (ii) DNA methylation can recruit methyl-CpG binding proteins that, in turn, are capable of recruiting chromatin-remodeling factors (reviewed in Klose & Bird, 2006).

As mentioned above, methylation can physically prevent transcription factors from binding to their recognition sequences (Watt & Molloy, 1988). For example, the glial fibrillary acidic protein gene is activated during astrocyte differentiation by the demethylation of a CpG dinucleotide located in a signal transducer and activator of transcription 3 binding element (STAT3) (Takizawa *et al.*, 2001). Although methylation normally leads to transcriptional silencing, methylation of repressor protein-binding elements, as in the imprinted insulin-like growth factor 2 (Igf2) gene, can increase its expression (Eden *et al.*, 2001; Murrell *et al.*, 2001).

Methylation signals can be interpreted directly by methyl-CpG-binding proteins that can alter gene transcription (Klose & Bird, 2006). In vertebrates, there are two families of methyl-CpG-binding proteins: the methyl-binding domain (MBD) family and the Kaiso family (reviewed in Bogdanovic & Veenstra, 2009). Five members of the MBD family have been identified: MeCP2, MBD1, MBD2, MBD3 and MBD4, although MBD3 presents a mutation on the MBD domain and is unable to bind methylated CpGs (Hendrich & Bird, 1998). Unlike the MBD members, the Kaiso family lacks an MBD, but binds to methyl-CpGs through a zinc finger domain (Prokhortchouk *et al.*, 2001). All members of the MBD family, except MBD4, have been shown to act as transcriptional repressors *in vitro* (Hendrich & Bird, 1998). In contrast, MBD4 has been associated with minimizing mutations at 5-methylcytosine and in DNA repair (Bellacosa *et al.*, 1999; Hendrich *et al.*, 1999).

A number of studies have shown that members of the MBD family play different roles throughout development and in disease. This is attributed to the capability of the MBD proteins to interact with repressing complexes such as Nucleosome Remodeling and histone Deacetylation (NuRD), RE1-silencing transcription factor (REST) and REST corepressor 1 (CoREST), as well as with various chromatin remodelers such as histone deacetylases (HDACs), Dnmt1, Polycomb, Brahma and Alpha thalassemia/mental retardation syndrome X-linked (ATRX) (Jones *et al.*, 1998; Nan *et al.*, 1998, 2007; Zhang *et al.*, 1999; Lunyak *et al.*, 2002; Fuks *et al.*, 2003; Kimura & Shiota, 2003; Harikrishnan *et al.*, 2005). The interaction of MBDs with HDAC complexes (Prokhortchouk *et al.*, 2001) contributes to condensing the chromatin into higher order structures that are transcriptionally silent (Ng *et al.*, 1999; Wade *et al.*, 1999; Zhang *et al.*, 1999; Feng & Zhang, 2001). Therefore, the MBDs provide a link between DNA methylation-mediated transcriptional repression, histone deacetylation and chromatin remodeling. Among the different histone post-translational modifications, acetylation on different residues of histone 3 and histone 4 as well as methylation on histone 3 lysine 4 (H3K4me) are normally associated with an open chromatin state, whereas histone 3 methylation on lysine 9 and lysine 27 (H3K9me and H3K27me) is associated with a closed chromatin state (Kouzarides, 2007). There are some cases in which DNA methylation has been associated with histone marks (Vaissiere *et al.*, 2008). For example, reduction of cytosine methylation leads to an increase of histone 3 acetylation on lysine 9 and lysine 14 (H3K9Ac and H3K14Ac), whereas H3K4 methylation (H3K4me) causes a decrease in H3K9me in mammals (Nguyen *et al.*, 2001; Bachman *et al.*, 2003). However, it is still unknown whether a functional relationship exists between DNA methylation and histone methylation to control gene expression.

## Methyl-CpG binding protein 2 and disease

**Fig. 3:**
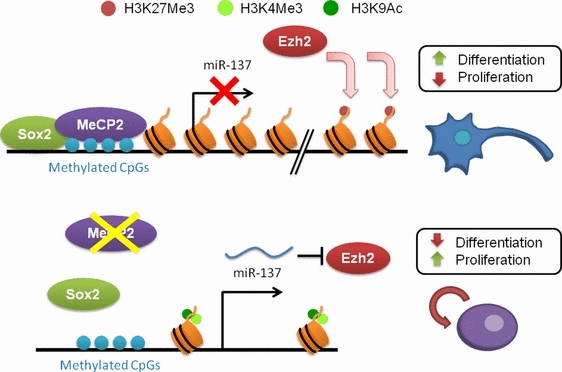
MeCP2 regulates differentiation through miR-137 and Ezh2. MeCP2 binds to methylated CpGs in a region upstream of miR-137 and, together with Sox2, represses miR-137 transcription. Ezh2, a target of miR-137, is not repressed and is able to establish H3K27me3 marks. This process enhances differentiation and inhibits proliferation of aNSCs. The absence of MeCP2 correlates with a gain of open chromatin marks (H3K9Ac and H3K4me3) in the area surrounding miR-137 and a loss of Sox2 in the regulatory region. Ezh2 is repressed by miR-137 that leads to a global loss of H3K27me3 marks; this enhances proliferation and inhibits differentiation of aNSCs (Szulwach *et al.*, 2010).

Methyl-CpG binding protein 2 was one of the first members of the MBD family to be discovered (Lewis *et al.*, 1992). Apart from the MBD domain, MeCP2 has a transcriptional repressor domain that is able to interact with other co-repressors (like deacetylase complexes), two nuclear localization signals and a tryptophan-tryptophan domain in the C-terminus that facilitates binding to nucleosomal DNA and is thought to mediate protein–protein interactions (reviewed in Bienvenu & Chelly, 2006; Fig. 3).

**Fig. 2:**
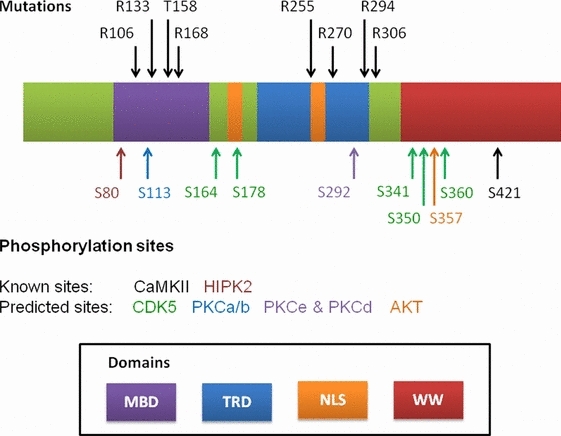
MeCP2 protein structure and common mutations associated with RTT. The MeCP2 protein domains, the most frequent mutations found in RTT and different phosphorylation sites are depicted. MeCP2 has different domains including a methyl CpG binding domain (MBD), a transcriptional repression domain (TRD), two nuclear localization signals (NLS) and a WW domain in the C-terminus. The black arrows (top) indicate the position of the eight most common mutations found in RTT; note that they locate preferentially to the MBD and TRD domains (Matijevic *et al.*, 2009). Known phosphorylation sites of MeCP2 are shown (bottom) for CaMKII (S421) and homeodomain-interacting kinase 2 (S80; Zhou *et al.*, 2006; Bracaglia *et al.*, 2009), as well as predicted phosphorylation sites for cyclin dependent kinase 5 (CDK5), PKCα/β, AKT, PKCε and PKCδ.

Methyl-CpG binding protein 2 is required for correct brain function and development. Loss of MeCP2 has been shown to delay neuronal maturation and synaptogenesis, and cause Rett syndrome (RTT; reviewed in Matijevic *et al.*, 2009). RTT is an X-linked, neurodevelopmental disorder that is characterized by a normal period of development from 6 to 18 months, followed by a subsequent loss of acquired speech and motor skills. Patients with RTT can also develop mental retardation, stereotyped hand movements, ataxia, seizures, microcephaly, autism and respiratory dysfunctions (Hagberg *et al.*, 1983). RTT is mostly caused by mutations on the *MeCP2* gene (Fig. 2), but mutations for cyclin-dependent kinase-like 5 (*CDKL5*) have also been associated with RTT (reviewed in Matijevic *et al.*, 2009; both genes are located on the X chromosome). Although CDKL5 has been shown to be able to phosphorylate MeCP2 and cause its release from the DNA (Mari *et al.*, 2005; Bertani *et al.*, 2006), a direct functional relationship between these two molecules in RTT is controversial (see below).

Methyl-CpG binding protein 2 brain levels in newborn mice are very low but increase significantly with development, reaching a maximum at 5 weeks, after which MeCP2 expression remains constant (Skene *et al.*, 2010). RTT has mostly been attributed to gene deregulation caused by a lack of MeCP2 expression in adult neurons (Luikenhuis *et al.*, 2004). This correlates with the fact that patients with RTT develop normally through the first 6–18 months of age after which they start presenting the characteristics of the disease (Hagberg *et al.*, 1983). This idea is supported by the phenotype of the MeCP2-null mice. MeCP2-null mice develop normally until 5–6 weeks of age, when they begin to show neurological symptoms, hind limb clasping and irregular breathing. They also present a reduction in weight and neuronal cell size, and normally die between 6 and 12 weeks (Chen *et al.*, 2001; Guy *et al.*, 2001).

Even though RTT has normally been attributed to neuronal MeCP2 deficiency (Luikenhuis *et al.*, 2004), several studies have begun to implicate non-neural cells in the development of the disease (Maezawa & Jin, 2010; Ballas *et al.*, 2009; Maezawa *et al.*, 2009; Table 1). Recently, it has been shown that MeCP2 is not only expressed in neurons, as previously thought (Skene *et al.*, 2010), but is also expressed in embryonic and adult stages in all glial cell types, including astrocytes, oligodendrocyte progenitor cells, oligodendrocytes and microglia. Analysis of the RTT mouse brain (Ballas *et al.*, 2009) shows loss of MeCP2 expression in astrocytes as well as low H3K9me3 levels, whereas H3Ac is enriched. Co-culture of neurons and astrocytes demonstrates that RTT astrocytes are unable to support normal dendritic morphology in wild-type (WT) hippocampal neurons, which show fewer and shorter dendrites. Culturing WT hippocampal neurons in astrocytic conditioned media from RTT astrocyte cultures results in aberrant dendritic morphology and a reduced neuronal density, whereas RTT neurons cultured in WT astrocytic conditioned media show normal dendritic morphology, suggesting that MeCP2 regulates the expression of soluble factors that are required for neuronal survival.

**Table 1:** MeCP2 deficiency in different cells

Cell type	Effect	Reference(s)
Neurons	Abnormal dendritic and axon development	Larimore *et al.* (2009)
Astrocytes	Unable to sustain proper neuron development	Ballas *et al.* (2009)Maezawa *et al.* (2009)
Microglia	Neurotoxic effect by excessive glutamate release	Maezawa & Jin (2010)

Another study has demonstrated that astrocytes from MeCP2^+/−^ mice show only 30% of the MeCP2 protein level with respect to WT, whereas MeCP2^+/−^ neurons show 70–80% compared with WT (Maezawa *et al.*, 2009). Astrocytic levels of MeCP2 do not correspond with the expected values for X chromosome inactivation, suggestive of a non-cell-autonomous mechanism of MeCP2 expression in astrocytes. In agreement with this, MeCP2 expression was reduced in a time-dependent manner in WT astrocytes when co-cultured with MeCP2^+/−^ astrocytes; additionally, astrocyte MeCP2 protein levels in MeCP2^+/−^ mice show a significant reduction with age. Inhibition of the gap junction communication resulted in a reduced spread of the MeCP2 deficiency and also rescued part of the phenotype. A more recent study by the same group showed that microglia also play an important role in the development of RTT (Maezawa & Jin, 2010). WT hippocampal neurons cultivated in conditioned media from very pure microglia cultures from MeCP2-null mice had shorter and thinner dendrites, with stunted arborization, whereas those grown in WT microglia conditioned media showed a normal phenotype. Consistently, neurons grown in MeCP2-null microglia conditioned media showed a reduced staining for MAP2 (Microtubule-associated protein 2, a dendritic marker), acetylated tubulin (reduction indicates microtubule disruption), PSD95 (postsynaptic density-95, a post-synaptic marker) and glutamate receptor interacting protein 1 (a scaffold protein in the post-synaptic compartment). All of this suggests that MeCP2-null microglia conditioned media damages post-synaptic elements of excitatory synapses (Maezawa & Jin, 2010). MeCP2-null microglia did not present an activated morphology or enhanced proliferation, indicative that the neurotoxic effect is not dependent on microglia activation. The authors report that this neurotoxic effect results from an excess of glutamate release from the MeCP2-null microglia and that it is released through channels of the gap junction. As in Maezawa *et al.* (2009), blocking the gap junction channels reduced the levels of released glutamate and rescued neuron morphology. Contrary to previous results (Ballas *et al.*, 2009), neurons grown in astrocytic conditioned media, from both WT and MeCP2-null mice, did not display damage in dendrites or synapses. For the effects seen in astrocytes, Maezawa & Jin (2010) suggest a loss of function in MeCP2-null astrocytes, which would be unable to support adequate dendritic development, whereas Ballas *et al.* (2009) suggest a gain of function in which astrocytes secrete a neurotoxic factor into the medium. Still further analysis would be necessary to determine the variability of soluble factors that are secreted by both astrocytes and microglia caused by an MeCP2 deficiency. Given that MeCP2 can regulate gene expression both positively and negatively (Chahrour *et al.*, 2008; Ben-Shachar *et al.*, 2009; Urdinguio *et al.*, 2010) (see below), it is probable that loss of MeCP2 could lead to the overexpression of neurotoxic factors as well a reduction in soluble factors that are necessary for neuronal survival. Overall, these results point out a previously unconsidered important role of glial cells in the development of the RTT.

## Methyl-CpG binding protein 2 targets genes in the central nervous system

Searching for possible MeCP2 targets to explain the RTT phenotype has not been an easy task. Until recently only two genes were confirmed as MeCP2 targets in the central nervous system: the brain-derived neurotrophic factor (*Bdnf*) and distal-les homeobox 5 (dlx5), which is on an imprinted region in chromosome 7 (reviewed in Chadwick & Wade, 2007). MeCP2 regulates *Bdnf* expression by binding to the promoter IV and repressing its transcription. This repression can be relieved by neuronal depolarization, which triggers Ca^2+^-dependent phosphorylation of MeCP2 and subsequent dissociation of MeCP2 from the *Bdnf* promoter IV, leading to increased *Bdnf* transcription (Chen *et al.*, 2003; Zhou *et al.*, 2006). However, contrary to what was expected, patients with RTT as well as mouse models of RTT show lower levels of *Bdnf* compared with healthy individuals and WT animals, respectively (Chang *et al.*, 2006). Many studies have been performed to elucidate how MeCP2 regulates *Bdnf* expression (Chen *et al.*, 2003; Wang *et al.*, 2006; Zhou *et al.*, 2006), which is key to understanding RTT as most of the RTT phenotypes can be attributed to a deregulation of *Bdnf* expression in the brain (Chang *et al.*, 2006; Table 2). Accordingly, *Bdnf* overexpression rescues the morphology of *MeCP2*-deficient neurons (Larimore *et al.*, 2009). Another study suggests that *Bdnf* downregulation in *MeCP2*-deficient mice may be mediated by repressor and co-repressor molecules like REST and CoREST (Abuhatzira *et al.*, 2007). MeCP2 is able to repress both REST and CoREST, which correlate with an overexpression of these two factors in *MeCP2*-deficient brain. REST and CoREST can bind between *Bdnf* promoters I and II, and repress *Bdnf* transcription. Thus, in the absence of MeCP2, *Bdnf* transcription is mainly regulated by REST/CoREST, which do not respond to neuronal activity, resulting in lower levels of Bdnf. In WT mice, MeCP2 binds to promoter IV of *Bdnf* and mediates neuronal activity-dependent transcription of *Bdnf*. Deregulation of REST/CoREST could also help to explain the expression pattern of other genes that have been found to be downregulated in MeCP2-deficient brains (Nomura *et al.*, 2008).

**Table 2:** Regulation of Bdnf by MeCP2

Mechanism	Model	Reference(s)
In the absence of neural activity, MeCP2 binds to Bdnf promoter IV and represses Bdnf expression	Rat hippocampal neurons	Chen *et al.* (2003)
Membrane depolarization causes calcium-dependent MeCP2 phosphorylation and subsequent release from Bdnf promoter IV		Zhou *et al.* (2006)
Loss of MeCP2 affects Bdnf secretion	Adult MeCP2-null mice (MeCP2^−/y^)	Wang *et al.* (2006)
Receptor of activated protein kinase C1 (Rack1) is able to dissociate MeCP2 from Bdnf promoter IV, leading to Bdnf activation	SH-SY5Y human neuroblastoma cells	He *et al.* (2010)
MeCP2-deficient brain presents lower levels of Bdnf	MeCP2-deficient human and mouse brains	Abuhatzira *et al.* (2007)
MeCP2 deficiency increases the expression of both REST and CoREST, which in turn downregulate Bdnf levels by binding between Bdnf promoters I and II	MeCP2-deficient human and mouse brains	Abuhatzira *et al.* (2007))

In addition to its well-accepted role as a transcriptional repressor, a considerable amount of data suggests a role for MeCP2 as a transcriptional activator (Chahrour *et al.*, 2008; Ben-Shachar *et al.*, 2009; Urdinguio *et al.*, 2010). Given that some of the phenotypes associated with RTT could be attributed to hypothalamic dysfunction, Chahrour *et al.* (2008) defined the hypothalamic gene profile of WT, *MeCP2*-null and MeCP2-overexpressing (MeCP2-tg) mice using microarray screening. This study identified 2582 genes that were misregulated in both MeCP2-tg and *MeCP2*-null mice. The authors identified 2148 genes that were upregulated in the MeCP2-tg mice and downregulated in the null mice, and only 377 that were downregulated in MeCP2-tg mice and upregulated in null mice. Among these genes are different neurotransmitters (e.g. corticotropin-releasing hormone and somatostatin) and neurotransmitter receptors (e.g. thyroid hormone receptor alpha and thyrotropin-releasing hormone receptor) that could help to explain the hypothalamic dysfunction. Also of note are different genes coding for regulatory proteins like *Bdnf*, *reelin*, *cAMP responsibe element binding protein 1* (*Creb1*) and *Creb3*, as well as chromatin remodelers like different *HDACs* and t*ransformation/transcription domain-associated protein* (*Trapp*), the latter being previously identified as an enriched transcript in the hypothalamus (Guerra-Crespo *et al.*, 2011; Table 3). Most of these genes were found to be upregulated by MeCP2, whereas only reelin and HDAC9 were found to be repressed by MeCP2. This shows that in the hypothalamus the majority of genes targeted by MeCP2 are positively regulated, which clashes with the idea of MeCP2 being primarily a repressor. However, bisulfite sequencing showed that genes repressed by MeCP2 presented a higher degree of methylation than genes activated by MeCP2. The fact that MeCP2 binds preferentially to methylated DNA (Skene *et al.*, 2010) suggests that, to promote gene expression, MeCP2 could be acting through another protein to bind and to activate its target genes. In agreement with this idea, chromatin immunoprecipitation assays show that MeCP2 is complexed with CREB1 in the promoter region of genes activated by MePC2, whereas a repressed gene did not show CREB1 enrichment. Interestingly, expressing both MeCP2 and CREB1, but not MeCP2 alone, enhances transcription of the MeCP2 target genes showing functional synergy. Thus, the effect of MeCP2 on gene expression depends on the proteins present at the promoter region of MePC2 target genes; when MeCP2 binds to co-repressors and HDACs it results in transcriptional repression. Nevertheless, a more thorough study of the methylation state in the genes activated by MeCP2 will help to shed more light on this issue, as well as looking for co-activators interacting directly with MeCP2 other than CREB1.

**Table 3:** MeCP2 targets and their function

Targets	Function	Reference(s)
Bdnf	Essential for neuronal survival, differentiation, synaptic plasticity and dendrite outgrowth	See Table 2
Dlx5	Imprinted gene, homeobox transcription factor, regulates the enzymes that synthesize GABA	Horike *et al.* (2005)
miR-137	Role in differentiation and proliferation, inhibits Ezh2	Szulwach *et al.* (2010)
miR-184	Role in differentiation and proliferation	Nomura *et al.* (2008)
		Liu *et al.* (2010)
REST	Repression of neurogenic genes	Abuhatzira *et al.* (2007)
In the hypothalamus	Chromatin remodelers – HDACs and Trapp	Chahrour *et al.* (2008)
	Neurotransmitters – Crh and Ssh	
	Receptors – Trhr and Thra	

Crh, corticotropin-releasing hormone; Dlx5, distal-les homeobox 5; Ssh, somatostatin; Trhr, thyrotropin-releasing hormone receptor; Thra, thyroid hormone receptor alpha; Trapp, transformation/transcription domain-associated protein.

A similar study performed in the cerebellum identified 1180 and 1102 altered genes in the MeCP2-tg and *MeCP2*-null mice, respectively (Ben-Shachar *et al.*, 2009). The fold change in most cases was subtle, between 1.2- and 2-fold, with the exception of the gene Prl2c2 (prolactin-like protein), which was overexpressed 12-fold in the MeCP2-tg mice with respect to WT mice. This gene was not reported in the previous hypothalamus screening (Chahrour *et al.*, 2008). As in the hypothalamus, MeCP2 activated the majority of the altered genes in the cerebellum. These results further support the idea that MeCP2 may act as a positive regulator of gene expression in the central nervous system and that this function is not restricted to the hypothalamus. Interestingly, whereas in the hypothalamus the screen found thousands of genes that were differentially expressed in the MeCP2-Tg and MeCP2-null mice (Chahrour *et al.*, 2008), in the cerebellum only hundreds of misregulated genes were identified (Ben-Shachar *et al.*, 2009). This difference indicates that, although some of the targets in different brain regions might be the same, there is still a considerable amount of genes that are differentially affected between different regions of the brain and suggests specific mechanisms of gene regulation that depend on the neuronal phenotype. This also supports the importance of epigenetic regulation and the greater impact it has on determining specificity for each tissue and cell type during development. It is also possible that MeCP2 might act preferentially as an activator on some cell types, whereas on others it might act as a repressor. Thus, looking at specific regions of the brain can provide more insights about MeCP2 targets and their regulation. However, it remains to be determined whether all of these genes are direct targets of MeCP2 or are regulated indirectly.

## Methyl-CpG binding protein 2 and microRNAs

Recently, the role of miRNAs in a number of pathologies has started to be elucidated. To decipher the impact of miRNAs downstream of MeCP2, Urdinguio *et al.* (2010) used a specific miRNA microarray to determine the expression profile in the *MeCP2*-null mice compared with WT mice. Sixty-five miRNAs showed differential expression between the two conditions. Among these, 45 were downregulated in *MeCP2*-null mice, whereas 18 were upregulated. Bisulfite sequencing and chromatin immunoprecipitation assays showed that, among four of the miRNAs identified, MeCP2 binds to methylated CpGs present on the 5′ regulatory region of three of them. MeCP2 binds to the methylated 5′ region of microRNA-29b (miR-29b), which is upregulated in *MeCP2*-null mice, correlating with the role of MeCP2 as a repressor for miR-29b. However, both miR-146a and miR-146b were downregulated in the MeCP2-null mice and had MeCP2 enrichment at their methylated 5′ ends, supporting a role for MeCP2 as a transcriptional activator. Both of these miRNAs target IRAK1 (interleukin-1 receptor-associated kinase; Taganov *et al.*, 2006), which is upregulated in RTT mouse models. Therefore, downregulation of miR-146a and miR-146b could lead to higher levels of IRAK1. Thus, enhanced interleukin-1β signaling in glia cells resulting from elevated IRAK levels might contribute to the release of neurotoxic agents (Thornton *et al.*, 2006) leading to neuronal death as previously described (Ballas *et al.*, 2009; Maezawa *et al.*, 2009; Maezawa & Jin, 2010).

The miRNAs prove a very interesting method of regulation as they can act at both the post-transcriptional level and the translational level (Filipowicz *et al.*, 2008). This suggests that the subtle changes in the mRNA levels of potential MeCP2 targets observed in different studies may not reflect the protein level. Thus, a proteomic approach in these models could certainly provide valuable information to fully understand RTT.

Other studies have also focused on the regulation of miRNAs by MeCP2. One of these miRNAs is miR-184, which is found on an imprinted region of chromosome 9 (Nomura *et al.*, 2008). Its expression is restricted to brain and testis and the paternal allele is the only one that is expressed in the brain. A CpG-rich region located downstream of the miR-184 transcript has been identified as hypermethylated only in brain (Nomura *et al.*, 2008). KCl treatment increases miR-184 expression similarly to *Bdnf* in cortical neurons. Chromatin immunoprecipitation assays have shown that after KCl treatment the enrichment of MeCP2 in the miR-184 CpG-rich region was reduced in the paternal allele only, correlating with miR-184 specific paternal expression. The MeCP2-deficient brain showed reduced miR-184 levels, which suggest a regulation similar to Bdnf. Interestingly, MBD1 also binds to this locus in the brain but, contrary to MeCP2, MBD1 deficiency causes miR-184 overexpression, as would be expected after the loss of a repressor (Liu *et al.*, 2010). These observations point out the distinct mechanisms used by the different members of the MBD family to regulate gene expression in the brain.

Furthermore, miR-184 results in an interesting MeCP2 target as it has been shown that this miRNA plays a role during neural development by inducing proliferation and inhibiting both neuronal and astrocytic differentiation (Liu *et al.*, 2010). miR-184 targets Numblike, which has been shown to increase neuronal differentiation. These results suggest that MeCP2 might also regulate neuronal differentiation by indirectly targeting Numblike through miR-184. Interestingly, miR-184 is expressed in the two known brain regions (hippocampus and subventricular zone) of adult neurogenesis *in vivo*. However, it remains to be determined whether adult neurogenesis is altered in the MePC2-deficient mice.

Another miRNA that is regulated by MeCP2 is miR-137 (Szulwach *et al.*, 2010). Overexpression of miR-137 enhances proliferation, whereas inhibition leads to a decreased proliferating capacity. Adult neural stem cell (aNSC) differentiation correlates with increased levels of miR-137. Consequently, MeCP2 deficiency promotes premature miR-137 expression before aNSC differentiation, consistent with MeCP2's role as a repressor (Matijevic *et al.*, 2009). Chromatin immunoprecipitation assays using MeCP2 antibodies show enrichment in the 5′-region of miR-137 relative to the null mice (MeCP2^−/y^) that correlates with increased methylated CpGs (Szulwach *et al.*, 2010). Analysis of different chromatin marks in MeCP2^−/y^ mice showed an increase in H3K4me3 and H3K9Ac in the region surrounding the miR-137 gene compared with WT mice (Szulwach *et al.*, 2010); both histone marks are associated with an open chromatin state. During aNSC differentiation the levels of H3K4Me and H3K9Ac are increased, supporting MeCP2 deficiency leading to the premature establishment of an open chromatin configuration. Additionally, an enrichment of Sox2 in the upstream region of miR-137 was observed, which is lost in the *MeCP2*-null mice, suggesting that Sox2 binding to DNA depends on its interaction with MeCP2. Accordingly, immunoprecipitation assays demonstrated Sox2–MeCP2 protein–protein interactions (Szulwach *et al.*, 2010).

The enhancer of Zeste homolog 2 (Ezh2), a H3K27 methyltransferase, has been reported as a putative target for miR-137 (Szulwach *et al.*, 2010). Ezh2 overexpression reduces cell proliferation and co-expression with miR-137 increases the number of differentiated neurons. Overexpression of miR-137 reduces Ezh2 protein levels; the same was observed in *MeCP2*-null mice where miR-137 is overexpressed. This effect is specific as reducing miR-137 recovered normal Ezh2 in MeCP2^−/y^ mice (Szulwach *et al.*, 2010). Thus, the overexpression of miR-137 and the decrease in Ezh2 correlated with an overall reduction of H3K27me3 in mouse aNSCs (Fig. 3 and Table 2). These results point out a cross-talk between different epigenetic processes to regulate gene expression leading to correct neural development and differentiation. Once again, it would be interesting to determine whether adult neurogenesis is altered in patients with RTT.

## Methyl-binding domains and cell differentiation

In addition to the necessary role of MeCP2 in mature neuronal and glial cells, it also has an important role during cell differentiation. MeCP2 promotes neuronal differentiation of neural stem cells while repressing astrocytic differentiation [induced by astrogenic factors like Leukemia inhibitory factor (Lif) and Bone morphogenetic protein 2 (BMP2); Tsujimura *et al.*, 2009]. Interestingly, the truncated mutant of MeCP2 (R168X, lacking the complete transcriptional repressor domain), identified in patients with RTT (Amir *et al.*, 1999), is unable to promote and suppress neuronal and astrocytic differentiation, respectively. This suggests a necessary role of this domain for the correct regulation by MeCP2.

In addition to MeCP2, other MBDs have also been implicated during cell differentiation processes (Li *et al.*, 2008; MacDonald *et al.*, 2010). The fibroblast growth factor 2 (FGF-2) is crucial for maintaining aNSCs in an undifferentiated state (Palmer *et al.*, 1995; Zheng *et al.*, 2004). Consequently, FGF-2 overexpression results in a significant reduction of neuronal differentiation. FGF-2 expression is regulated negatively by MBD1 that binds to a specific CpG-rich region on the mouse and rat *FGF-2* promoters (Li *et al.*, 2008). Accordingly, *Mbd1*^−/−^ mice show increased levels of FGF-2 that correlate with impaired aNSC differentiation and adult neurogenesis. Depletion of *Mbd1* using RNA interference results in a neuronal differentiation percentage that is significantly reduced from aNSCs. These results corroborate the crucial role of MBD1 in neuronal differentiation.

In the adult olfactory epithelium, the olfactory receptor neurons (ORNs) are in continuous replacement (Murdoch & Roskams, 2007). *Mbd2* has two alternative splice variants: MBD2a and MBD2b (Hendrich & Bird, 1998). MBD2a is found predominantly in the mature ORNs, whereas MBD2b is found in progenitors and immature post-mitotic neurons. Consistently, the *Mbd2*-null mice exhibit an increase in progenitor cell proliferation and a reduced number of mature ORNs (MacDonald *et al.*, 2010). In contrast, MeCP2 expression increases ORN maturation. *MeCP2*-null mice show an increased number of immature ORNs with no significant change in the number of progenitors or mature ORNs. Specific Dnmts and HDACs are present in the different stages of ORN differentiation (MacDonald *et al.*, 2005). Cycling progenitor cells express high levels of Dnmt3b and HDAC1, whereas they are downregulated in post-mitotic neurons, which express Dnmt3a and HDAC2. As neurons mature, Dnmt3a and HDAC2 expression levels are downregulated, whereas HDAC1 is expressed again. Therefore, in addition to MeCP2, MBD2 could regulate pivotal stages of ORN development via interaction with Dnmts, HDACs and MeCP2 as they are co-expressed at different stages of ORN maturation and have been reported to co-immunoprecipitate (MacDonald *et al.*, 2005, 2010).

In addition to Dnmt–HDAC interaction, recent evidence shows the existence of specific interactions between Dnmts and histone methyltransferases that incorporate repressive histone marks. Dnmt3 together with the H3K9 methyltranferase G9A cooperatively ensures proper neurogenesis in zebrafish (Rai *et al.*, 2010). Whether the Dnmt3 family in mammals displays a similar Dnmt–histone methyltransferase relationship will be a matter for future investigations.

## Signals controlling methyl-CpG binding protein 2 gene expression and function

A precise regulation of MeCP2 levels is needed as both deficient and overexpressing brains exhibit RTT-like phenotypes (Ramocki *et al.*, 2009). Quantification studies have estimated 16 × 10^6^ molecules of MeCP2 per nucleus in neurons (Skene *et al.*, 2010). The estimated number of methylated CpG sites is about 40 × 10^6^ sites per nucleus, meaning that there are enough MeCP2 molecules to bind approximately half of the CpG sites. Histone H1 is present at a ratio of one molecule per nucleosome, although in neurons this relationship is reduced to one H1 molecule for every two nucleosomes (Pearson *et al.*, 1984). MeCP2 can compete with H1 for binding to methylated chromatin, functioning as a linker histone (Nan *et al.*, 1997). In agreement with this, *MeCP2*-null mouse brain shows a twofold increase in H1, supporting the idea that, in the brain, MeCP2 binds half of the nucleosomes and H1 binds the other half (Skene *et al.*, 2010). This suggests that MeCP2 deficiency could also lead to an overall chromatin decondensation that would result in gene deregulation as seen in previous screens.

The MeCP2 gene is known to have multiple polyadenylation sites (Reichwald *et al.*, 2000); the longer transcript (approximately 10 kb) is predominantly transcribed in the brain and its 3′ untranslated region contains target motifs for different miRNAs. One of these sites is for miR-132, which plays a role in neurite outgrowth mediated by Bdnf (Vo *et al.*, 2005). Overexpression of miR-132, as well as treatment with Forskolin and KCl (which induce miR-132 expression through CREB), in primary cortical neurons decreased MeCP2 protein levels (Klein *et al.*, 2007). Bdnf levels are increased both by MeCP2 overexpression and by blocking miR-132 with an antisense oligonucleotide. MeCP2 overexpression induces *Bdnf* expression, which in turn increases miR-132 transcription that downregulates MeCP2 protein production. This suggests a homeostatic mechanism for maintaining correct MeCP2 levels (Klein *et al.*, 2007); however, little is known about additional factors regulating the expression of MeCP2 gene in the brain.

Few reports are available regarding extracellular signals regulating MeCP2 function. Recently, it has been shown that MeCP2 is phosphorylated by Calcium/calmodulin-dependent protein kinase II (CaMKII) on Ser421 in response to neuronal depolarization and Bdnf (Zhou *et al.*, 2006). This phosphorylation releases MeCP2 from the promoter IV of *Bdnf* resulting in increased Bdnf expression (Chen *et al.*, 2003). Consistent with this, expression of an MeCP2 S421A mutant prevents neurite arborization and reduces Bdnf expression (Tao *et al.*, 2009), indicating that MeCP2 phosphorylation at this residue promotes MeCP2 release from its targets allowing their expression. Although MeCP2 is expressed in many adult tissues it is phosphorylated at Ser421 almost exclusively in the adult brain (Zhou *et al.*, 2006), suggesting that phosphorylation of MeCP2 at this residue is required for homeostatic brain functions. MeCP2 phosphorylation at the amino-terminus also modulates its DNA binding and silencing activity. In contrast to Ser421, phosphorylation at Ser80 is required for MeCP2 binding to DNA and correct regulation of target genes like gene trap locus 2 (*Gtl2*) and proopiomelanocortin (*Pomc*) among others (Tao *et al.*, 2009). Transgenic knock-in mice carrying the S80A mutation show reduced MeCP2 binding to target genes and consequently aberrant gene expression (Tao *et al.*, 2009). The homeodomain-interacting kinase 2 has been shown to phosphorylate MeCP2 at Ser80 (Bracaglia *et al.*, 2009). Accordingly, both the homeodomain-interacting kinase 2-deficient mice and S80A knock-in mice show locomotion defects, suggesting that phosphorylation at this residue by HIP2K regulates MeCP2 activity *in vivo* (Zhang *et al.*, 2007). Interestingly, although signals that promote neuronal survival like neuronal activity and calcium fluxes lead to Ser80 dephosphorylation and Ser421 phosphorylation, MeCP2 phosphorylation by HIP2K at Ser80 has been associated with cell death (Bracaglia *et al.*, 2009). Thus, it remains to be determined whether phosphorylation at this site promotes or represses the expression of pro-apoptotic or antiapoptotic genes, respectively; and also whether the phenotypes observed in the S80A knock-in mice and homeodomain-interacting kinase 2-deficient mice result from altered neural cell death. Nevertheless, it seems that both Ser80 dephosphorylation and Ser421 phosphorylation are required to dissociate MeCP2 from DNA in response to neuronal activity and calcium influx.

**Fig. 4:**
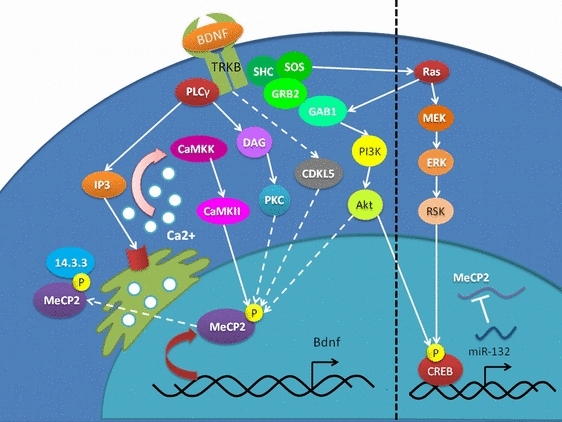
Regulation of MeCP2 expression by Bdnf. Post-translational regulation of MeCP2 – MeCP2 is phosphorylated by CaMKII in response to neuron depolarization and Bdnf signaling (Zhou *et al.*, 2006). MeCP2 could also be phosphorylated by other kinases downstream of the Bdnf signaling pathway (dashed lines) resulting in interactions with the 14.3.3 proteins, thus regulating MeCP2 localization and function. Post-transcriptional regulation of MeCP2 – MeCP2's 3′ untranslated region contains sites for miR-132, which is activated by CREB. CREB is also downstream of the Bdnf signaling pathway, suggesting the presence of a regulatory loop between Bdnf and MeCP2. AKT, v-akt murine thymoma viral oncogene homolog 1; CAMKK, calcium/calmodulin-dependent protein kinase kinase; DAG, diacylglycerol; ERK/MAPK, mitogen activated protein kinase; GAB1, GRB2 associated binder; GRB2, growth factor receptor bound protein 2; IP3, inositol triphosphate; MEK/MAPKK, mitogen activated protein kinase kinase, PI3K, phosphoinositide-3-kinase; PKC, protein kinase C; PLCγ, Phospholipase C gamma 1; Ras, rat sarcoma viral oncogene homolog; RSK, ribosomal protein S6 kinase; SHC, Src homology 2 domain containing transforming protein 1; SOS, son of sevenless.

As mentioned above, mutations in CDKL5 have been identified in patients with RTT and other encephalopathies (Matijevic *et al.*, 2009). The fact that MeCP2–CDKL5 protein–protein interactions have been shown by two independent groups, and that mutations in CDKL5 found in patients with RTT alter its kinase activity and cellular distribution (Mari *et al.*, 2005), suggest that these naturally occurring mutations alter the MeCP2–CDKL5 interaction and point out a functional role for these molecules in RTT development. Although it is not yet clear whether MeCP2 is a direct target of CDKL5, Chen *et al.* (2010) have shown that CDKL5 is required for normal neurite outgrowth, a process that is altered in RTT neurons (Jellinger *et al.*, 1988). Interestingly, Bdnf regulates neurite outgrowth by promoting CDKL5 kinase activity leading to ras-related C3 botulinum toxin substrate 1 (rac-1) activation and neurite arborization (Chen *et al.*, 2010). Thus, we propose that, during normal development, neuronal activity and other extracellular signals promote MeCP2 dephosphorylation at Ser80 and phosphorylation at Ser421 alleviating MeCP2 repression of the *Bdnf* gene. Bdnf is then produced, secreted and through its receptor initiates an autocrine loop that further inhibits MeCP2 function at two levels: (i) preventing MeCP2 DNA binding through phosphorylation mediated by CDKL5 and/or CAMKII and (ii) reducing MeCP2 levels by inducing miR-132 via CREB, thus ensuring the expression of *Bdnf* and other genes involved in neurite outhgrowth (Fig. 4).

Brain-derived neurotrophic factor, through its specific receptor Neurotrophic tyrosine kinase receptor type 2 (TrkB), activates distinct signal transduction pathways [protein kinase C (PKC), v-akt murine thymoma viral oncogene homolog 1 (AKT) and mitogen-activated protein kinase (MAPK)] that regulate neural functions (Yoshii & Constantine-Paton, 2010). MeCP2 protein contains different domains that may be targeted by these pathways as demonstrated by a bioinformatic analysis (Motif Scan) performed in our laboratory. This analysis revealed potential phosphorylation sites for cyclin-dependent kinase 5 (CDK5) in the central region of the molecule (Ser164, Ser178, Ser341, Ser350 and Ser360), as well as putative sites for different members of the PKC family – at the amino-terminus for PKCα/β (Ser113) and in the central region for PKCε and PKCδ (Ser292; Fig. 3). Interestingly, a potential AKT phosphorylation site (Ser357) is located within a putative MeCP2–14.3.3 interaction domain (SPKGRSSS^357^ASSPPKK). This domain also contains Ser350 and Ser360, which could be phosphorylated by CDKL5 (Fig. 2). 14.3.3 proteins regulate protein function by steric competition or by modulating cellular distribution of its targets (Mhawech, 2005). The interaction of 14.3.3 proteins with their molecular targets is regulated by phosphorylation events (Deribe *et al.*, 2010). Both AKT- and PKC-mediated phosphorylation of BCL2-associated agonist of cell death (Bad) or v-raf-1 murine leukemia viral oncogene homolog 1 (Raf), among other targets, regulates cell survival and proliferation, respectively. Moreover, cyclin-dependent kinases regulate the cell cycle in part by phosphorylating 14.3.3 partners (Morrison, 2009). Taken together, it is possible that Bdnf prevents MeCP2 transcriptional functions by promoting its interaction with 14.3.3 proteins through a mechanism involving MeCP2 phosphorylation on Ser357 by AKT and/or on Ser350 and Ser360 by CDKL5, thus favoring neurite outgrowth (Fig. 4).

Several of the mutations observed in MeCP2 associated with RTT are located near putative phosphorylation sites – mutation of the Arg110 residue could alter the phosphorylation of Ser113 by PKCα/β, mutation of Arg294 might affect phosphorylation of Ser292 by PKCε or PKCδ, and mutations of Arg168 could impair Ser164 phosphorylation by CDKL5 (Fig. 4). This strongly suggests that mutations in MeCP2 found in RTT could impair Bdnf-mediated MeCP2 regulation resulting in abnormal neurite outgrowth. However, it remains to be determined whether MeCP2 is phosphorylated by any of these kinases in response to Bdnf and whether this modulates MeCP2-dependent gene expression.

## Conclusions

Epigenetic regulation of chromatin architecture plays an essential role in brain development and synaptic plasticity, particularly in brain areas related to cognitive and emotional processes. Although the gross anatomy of the brain is largely established at birth, cognitive development during the post-natal period depends upon reorganization in synaptic connectivity driven by sensory experience (Wiesel, 1982). Several neurodevelopmental cognition disorders manifest during this period, suggesting that they might be primarily diseases of synaptic maturation (Zoghbi, 2003). Most major mental illnesses have been proposed to result from multiple risk genes, which supports a stressful environment triggering epigenetic changes in the expression of normal genes (Murgatroyd *et al.*, 2009). Both inherited risk genes as well as abnormalities in epigenetic regulation of normal genes have been implicated in the pathophysiology of many neurodevelopmental or psychiatric disorders. The examples discussed here highlight the importance of methylomics, the regulation of gene expression by methylating DNA and histones, as a key epigenetic mechanism in RTT.

Further mechanistic understanding of RTT depends on identifying and validating additional genes responsible for this disorder, specially focusing on the targets that MeCP2 might have on different brain cell types and distinct neuronal phenotypes. Accordingly, a recent study demonstrates that MeCP2 regulates L1 retrotransposition specifically in neurons (Muotri *et al.*, 2010). These observations provide a new mechanism by which loss of MeCP2 might result in altered gene expression leading to disease. However, additional studies are needed to evaluate the effects of MeCP2 deficiency in glial cells as well as at earlier stages of neuronal development, and not just focusing on mature neurons. Also, determining the various levels of cross-talk between different epigenetic mechanisms would help understand the processes leading to RTT and other neurodevelopmental diseases. It would be interesting to determine whether single nucleotide polymorphisms, long sequence polymorphisms or copy number variations in the genes encoding for DNA methyl transferases, histone-modifying enzymes or chromatin-remodeling complexes play a role in the development of neurodevelopmental, neurodegenerative or psychiatric disorders.

## Abbreviations

aNSC: adult neural stem cell
Bdnf: brain-derived neurotrophic factor
CDKL5: cyclin-dependent kinase-like 5
CoREST: REST corepressor 1
CpG: 5′-CG-3′
Dnmt: DNA methyltransferase
Ezh2: enhancer of Zeste homolog 2
FGF-2: fibroblast growth factor 2
HDAC: histone deacetylase
MBD: methyl binding domain
MeCP2: methyl-CpG binding protein 2
MeCP2-tg: MeCP2-overexpressing
miRNA or miR: microRNA
ORN: olfactory receptor neuron
PKC: Protein Kinase C
REST: RE1-silencing transcription factor
RTT: Rett syndrome
Sox2: Sex determining region Y-box 2
WT: wild-type

## References

[b1] Abuhatzira L, Makedonski K, Kaufman Y, Razin A, Shemer R (2007). MeCP2 deficiency in the brain decreases BDNF levels by REST/CoREST-mediated repression and increases TRKB production. Epigenetics.

[b2] Amir RE, Van den Veyver IB, Wan M, Tran CQ, Francke U, Zoghbi HY (1999). Rett syndrome is caused by mutations in X-linked MECP2, encoding methyl-CpG-binding protein 2. Nat. Genet..

[b3] Antequera F, Bird A (1993). Number of CpG islands and genes in human and mouse. Proc. Natl Acad. Sci. USA.

[b4] Bachman KE, Park BH, Rhee I, Rajagopalan H, Herman JG, Baylin SB, Kinzler KW, Vogelstein B (2003). Histone modifications and silencing prior to DNA methylation of a tumor suppressor gene. Cancer Cell.

[b5] Ballas N, Lioy DT, Grunseich C, Mandel G (2009). Non-cell autonomous influence of MeCP2-deficient glia on neuronal dendritic morphology. Nat. Neurosci..

[b6] Becker PB, Ruppert S, Schutz G (1987). Genomic footprinting reveals cell type-specific DNA binding of ubiquitous factors. Cell.

[b7] Bellacosa A, Cicchillitti L, Schepis F, Riccio A, Yeung AT, Matsumoto Y, Golemis EA, Genuardi M, Neri G (1999). MED1, a novel human methyl-CpG-binding endonuclease, interacts with DNA mismatch repair protein MLH1. Proc. Natl Acad. Sci. USA.

[b8] Ben-Shachar S, Chahrour M, Thaller C, Shaw CA, Zoghbi HY (2009). Mouse models of MeCP2 disorders share gene expression changes in the cerebellum and hypothalamus. Hum. Mol. Genet..

[b9] Berger SL (2007). The complex language of chromatin regulation during transcription. Nature.

[b10] Bertani I, Rusconi L, Bolognese F, Forlani G, Conca B, De Monte L, Badaracco G, Landsberger N, Kilstrup-Nielsen C (2006). Functional consequences of mutations in CDKL5, an X-linked gene involved in infantile spasms and mental retardation. J. Biol. Chem..

[b11] Bienvenu T, Chelly J (2006). Molecular genetics of Rett syndrome: when DNA methylation goes unrecognized. Nat. Rev. Genet..

[b12] Bird AP (1978). Use of restriction enzymes to study eukaryotic DNA methylation: II. The symmetry of methylated sites supports semi-conservative copying of the methylation pattern. J. Mol. Biol..

[b13] Bird AP (1986). CpG-rich islands and the function of DNA methylation. Nature.

[b14] Bogdanovic O, Veenstra GJ (2009). DNA methylation and methyl-CpG binding proteins: developmental requirements and function. Chromosoma.

[b15] Bonfils C, Beaulieu N, Chan E, Cotton-Montpetit J, MacLeod AR (2000). Characterization of the human DNA methyltransferase splice variant Dnmt1b. J. Biol. Chem..

[b16] Bracaglia G, Conca B, Bergo A, Rusconi L, Zhou Z, Greenberg ME, Landsberger N, Soddu S, Kilstrup-Nielsen C (2009). Methyl-CpG-binding protein 2 is phosphorylated by homeodomain-interacting protein kinase 2 and contributes to apoptosis. EMBO Rep..

[b17] Chadwick LH, Wade PA (2007). MeCP2 in Rett syndrome: transcriptional repressor or chromatin architectural protein?. Curr. Opin. Genet. Dev..

[b18] Chahrour M, Jung SY, Shaw C, Zhou X, Wong ST, Qin J, Zoghbi HY (2008). MeCP2, a key contributor to neurological disease, activates and represses transcription. Science.

[b19] Chang Q, Khare G, Dani V, Nelson S, Jaenisch R (2006). The disease progression of Mecp2 mutant mice is affected by the level of BDNF expression. Neuron.

[b20] Chen RZ, Pettersson U, Beard C, Jackson-Grusby L, Jaenisch R (1998). DNA hypomethylation leads to elevated mutation rates. Nature.

[b21] Chen RZ, Akbarian S, Tudor M, Jaenisch R (2001). Deficiency of methyl-CpG binding protein-2 in CNS neurons results in a Rett-like phenotype in mice. Nat. Genet..

[b22] Chen WG, Chang Q, Lin Y, Meissner A, West AE, Griffith EC, Jaenisch R, Greenberg ME (2003). Derepression of BDNF transcription involves calcium-dependent phosphorylation of MeCP2. Science.

[b23] Chen Q, Zhu YC, Yu J, Miao S, Zheng J, Xu L, Zhou Y, Li D, Zhang C, Tao J, Xiong ZQ (2010). CDKL5, a protein associated with rett syndrome, regulates neuronal morphogenesis via Rac1 signaling. J. Neurosci..

[b24] Chew JL, Loh YH, Zhang W, Chen X, Tam WL, Yeap LS, Li P, Ang YS, Lim B, Robson P, Ng HH (2005). Reciprocal transcriptional regulation of Pou5f1 and Sox2 via the Oct4/Sox2 complex in embryonic stem cells. Mol. Cell. Biol..

[b25] Clapier CR, Cairns BR (2009). The biology of chromatin remodeling complexes. Annu. Rev. Biochem..

[b26] Colot V, Maloisel L, Rossignol JL (1996). Interchromosomal transfer of epigenetic states in Ascobolus: transfer of DNA methylation is mechanistically related to homologous recombination. Cell.

[b27] Deribe YL, Pawson T, Dikic I (2010). Post-translational modifications in signal integration. Nat. Struct. Mol. Biol..

[b28] Eden S, Constancia M, Hashimshony T, Dean W, Goldstein B, Johnson AC, Keshet I, Reik W, Cedar H (2001). An upstream repressor element plays a role in Igf2 imprinting. EMBO J..

[b29] Feng Q, Zhang Y (2001). The MeCP1 complex represses transcription through preferential binding, remodeling, and deacetylating methylated nucleosomes. Genes Dev..

[b30] Filipowicz W, Bhattacharyya SN, Sonenberg N (2008). Mechanisms of post-transcriptional regulation by microRNAs: are the answers in sight?. Nat. Rev. Genet..

[b31] Fuks F, Hurd PJ, Wolf D, Nan X, Bird AP, Kouzarides T (2003). The methyl-CpG-binding protein MeCP2 links DNA methylation to histone methylation. J. Biol. Chem..

[b32] Goll MG, Bestor TH (2005). Eukaryotic cytosine methyltransferases. Annu. Rev. Biochem..

[b33] Guerra-Crespo M, Pérez-Monter C, Janga SC, Castillo-Ramirez S, Gutierrez-Rios RM, Joseph-Bravo P, Pérez-Martínez L, Charli JL (2011). Transcriptional profiling of fetal hypothalamic TRH neurons. BMC Genomics.

[b34] Guy J, Hendrich B, Holmes M, Martin JE, Bird A (2001). A mouse Mecp2-null mutation causes neurological symptoms that mimic Rett syndrome. Nat. Genet..

[b35] Hagberg B, Aicardi J, Dias K, Ramos O (1983). A progressive syndrome of autism, dementia, ataxia, and loss of purposeful hand use in girls: Rett's syndrome: report of 35 cases. Ann. Neurol..

[b36] Harikrishnan KN, Chow MZ, Baker EK, Pal S, Bassal S, Brasacchio D, Wang L, Craig JM, Jones PL, Sif S, El-Osta A (2005). Brahma links the SWI/SNF chromatin-remodeling complex with MeCP2-dependent transcriptional silencing. Nat. Genet..

[b37] Hashimshony T, Zhang J, Keshet I, Bustin M, Cedar H (2003). The role of DNA methylation in setting up chromatin structure during development. Nat. Genet..

[b38] He DY, Neasta J, Ron D (2010). Epigenetic regulation of BDNF expression via the scaffolding protein RACK1. J. Biol. Chem..

[b39] Hendrich B, Bird A (1998). Identification and characterization of a family of mammalian methyl-CpG binding proteins. Mol. Cell. Biol..

[b40] Hendrich B, Hardeland U, Ng HH, Jiricny J, Bird A (1999). The thymine glycosylase MBD4 can bind to the product of deamination at methylated CpG sites. Nature.

[b41] Holliday R, Pugh JE (1975). DNA modification mechanisms and gene activity during development. Science.

[b42] Horike S, Cai S, Miyano M, Cheng JF, Kohwi-Shigematsu T (2005). Loss of silent-chromatin looping and impaired imprinting of DLX5 in Rett syndrome. Nat. Genet..

[b43] Houbaviy HB, Murray MF, Sharp PA (2003). Embryonic stem cell-specific MicroRNAs. Dev. Cell.

[b44] Jaenisch R, Schnieke A, Harbers K (1985). Treatment of mice with 5-azacytidine efficiently activates silent retroviral genomes in different tissues. Proc. Natl Acad. Sci. USA.

[b45] Jellinger K, Armstrong D, Zoghbi HY, Percy AK (1988). Neuropathology of Rett syndrome. Acta Neuropathol..

[b46] Jones PL, Veenstra GJ, Wade PA, Vermaak D, Kass SU, Landsberger N, Strouboulis J, Wolffe AP (1998). Methylated DNA and MeCP2 recruit histone deacetylase to repress transcription. Nat. Genet..

[b47] Jurata LW, Thomas JB, Pfaff SL (2000). Transcriptional mechanisms in the development of motor control. Curr. Opin. Neurobiol..

[b48] Kamakaka RT, Biggins S (2005). Histone variants: deviants?. Genes Dev..

[b49] Keshet I, Lieman-Hurwitz J, Cedar H (1986). DNA methylation affects the formation of active chromatin. Cell.

[b50] Kimura H, Shiota K (2003). Methyl-CpG-binding protein, MeCP2, is a target molecule for maintenance DNA methyltransferase, Dnmt1. J. Biol. Chem..

[b51] Klein ME, Lioy DT, Ma L, Impey S, Mandel G, Goodman RH (2007). Homeostatic regulation of MeCP2 expression by a CREB-induced microRNA. Nat. Neurosci..

[b52] Klose RJ, Bird AP (2006). Genomic DNA methylation: the mark and its mediators. Trends Biochem. Sci..

[b53] Kouzarides T (2007). Chromatin modifications and their function. Cell.

[b54] Kuwabara T, Hsieh J, Muotri A, Yeo G, Warashina M, Lie DC, Moore L, Nakashima K, Asashima M, Gage FH (2009). Wnt-mediated activation of NeuroD1 and retro-elements during adult neurogenesis. Nat. Neurosci..

[b55] Larimore JL, Chapleau CA, Kudo S, Theibert A, Percy AK, Pozzo-Miller L (2009). Bdnf overexpression in hippocampal neurons prevents dendritic atrophy caused by Rett-associated MECP2 mutations. Neurobiol. Dis..

[b56] Lee SK, Pfaff SL (2001). Transcriptional networks regulating neuronal identity in the developing spinal cord. Nat. Neurosci..

[b57] Leonhardt H, Page AW, Weier HU, Bestor TH (1992). A targeting sequence directs DNA methyltransferase to sites of DNA replication in mammalian nuclei. Cell.

[b58] Lewis JD, Meehan RR, Henzel WJ, Maurer-Fogy I, Jeppesen P, Klein F, Bird A (1992). Purification, sequence, and cellular localization of a novel chromosomal protein that binds to methylated DNA. Cell.

[b59] Li E, Beard C, Jaenisch R (1993). Role for DNA methylation in genomic imprinting. Nature.

[b60] Li X, Barkho BZ, Luo Y, Smrt RD, Santistevan NJ, Liu C, Kuwabara T, Gage FH, Zhao X (2008). Epigenetic regulation of the stem cell mitogen Fgf-2 by Mbd1 in adult neural stem/progenitor cells. J. Biol. Chem..

[b61] Liu C, Teng ZQ, Santistevan NJ, Szulwach KE, Guo W, Jin P, Zhao X (2010). Epigenetic regulation of miR-184 by MBD1 governs neural stem cell proliferation and differentiation. Cell Stem Cell.

[b62] Luikenhuis S, Giacometti E, Beard CF, Jaenisch R (2004). Expression of MeCP2 in postmitotic neurons rescues Rett syndrome in mice. Proc. Natl Acad. Sci. USA.

[b63] Lunyak VV, Burgess R, Prefontaine GG, Nelson C, Sze SH, Chenoweth J, Schwartz P, Pevzner PA, Glass C, Mandel G, Rosenfeld MG (2002). Corepressor-dependent silencing of chromosomal regions encoding neuronal genes. Science.

[b64] MacDonald JL, Gin CS, Roskams AJ (2005). Stage-specific induction of DNA methyltransferases in olfactory receptor neuron development. Dev. Biol..

[b65] MacDonald JL, Verster A, Berndt A, Roskams AJ (2010). MBD2 and MeCP2 regulate distinct transitions in the stage-specific differentiation of olfactory receptor neurons. Mol. Cell. Neurosci..

[b66] Maezawa I, Jin LW (2010). Rett syndrome microglia damage dendrites and synapses by the elevated release of glutamate. J. Neurosci..

[b67] Maezawa I, Swanberg S, Harvey D, LaSalle JM, Jin LW (2009). Rett syndrome astrocytes are abnormal and spread MeCP2 deficiency through gap junctions. J. Neurosci..

[b68] Mari F, Azimonti S, Bertani I, Bolognese F, Colombo E, Caselli R, Scala E, Longo I, Grosso S, Pescucci C, Ariani F, Hayek G, Balestri P, Bergo A, Badaracco G, Zappella M, Broccoli V, Renieri A, Kilstrup-Nielsen C, Landsberger N (2005). CDKL5 belongs to the same molecular pathway of MeCP2 and it is responsible for the early-onset seizure variant of Rett syndrome. Hum. Mol. Genet..

[b69] Matijevic T, Knezevic J, Slavica M, Pavelic J (2009). Rett syndrome: from the gene to the disease. Eur. Neurol..

[b70] Mattick JS, Makunin IV (2005). Small regulatory RNAs in mammals. Hum. Mol. Genet..

[b71] Mhawech P (2005). 14-3-3 proteins – an update. Cell Res..

[b72] Miranda TB, Jones PA (2007). DNA methylation: the nuts and bolts of repression. J. Cell. Physiol..

[b73] Morrison DK (2009). The 14-3-3 proteins: integrators of diverse signaling cues that impact cell fate and cancer development. Trends Cell Biol..

[b74] Muotri AR, Marchetto MC, Coufal NG, Oefner R, Yeo G, Nakashima K, Gage FH (2010). L1 retrotransposition in neurons is modulated by MeCP2. Nature.

[b75] Murdoch B, Roskams AJ (2007). Olfactory epithelium progenitors: insights from transgenic mice and in vitro biology. J. Mol. Histol..

[b76] Murgatroyd C, Patchev AV, Wu Y, Micale V, Bockmuhl Y, Fischer D, Holsboer F, Wotjak CT, Almeida OF, Spengler D (2009). Dynamic DNA methylation programs persistent adverse effects of early-life stress. Nat. Neurosci..

[b77] Murrell A, Heeson S, Bowden L, Constancia M, Dean W, Kelsey G, Reik W (2001). An intragenic methylated region in the imprinted Igf2 gene augments transcription. EMBO Rep..

[b78] Nan X, Campoy FJ, Bird A (1997). MeCP2 is a transcriptional repressor with abundant binding sites in genomic chromatin. Cell.

[b79] Nan X, Ng HH, Johnson CA, Laherty CD, Turner BM, Eisenman RN, Bird A (1998). Transcriptional repression by the methyl-CpG-binding protein MeCP2 involves a histone deacetylase complex. Nature.

[b80] Nan X, Hou J, Maclean A, Nasir J, Lafuente MJ, Shu X, Kriaucionis S, Bird A (2007). Interaction between chromatin proteins MECP2 and ATRX is disrupted by mutations that cause inherited mental retardation. Proc. Natl Acad. Sci. USA.

[b81] Ng HH, Zhang Y, Hendrich B, Johnson CA, Turner BM, Erdjument-Bromage H, Tempst P, Reinberg D, Bird A (1999). MBD2 is a transcriptional repressor belonging to the MeCP1 histone deacetylase complex. Nat. Genet..

[b82] Nguyen CT, Gonzales FA, Jones PA (2001). Altered chromatin structure associated with methylation-induced gene silencing in cancer cells: correlation of accessibility, methylation, MeCP2 binding and acetylation. Nucleic Acids Res..

[b83] Nomura T, Kimura M, Horii T, Morita S, Soejima H, Kudo S, Hatada I (2008). MeCP2-dependent repression of an imprinted miR-184 released by depolarization. Hum. Mol. Genet..

[b84] Okano M, Bell DW, Haber DA, Li E (1999). DNA methyltransferases Dnmt3a and Dnmt3b are essential for de novo methylation and mammalian development. Cell.

[b85] Palmer TD, Ray J, Gage FH (1995). FGF-2-responsive neuronal progenitors reside in proliferative and quiescent regions of the adult rodent brain. Mol. Cell. Neurosci..

[b86] Panning B, Jaenisch R (1996). DNA hypomethylation can activate Xist expression and silence X-linked genes. Genes Dev..

[b87] Pearson EC, Bates DL, Prospero TD, Thomas JO (1984). Neuronal nuclei and glial nuclei from mammalian cerebral cortex. Nucleosome repeat lengths, DNA contents and H1 contents. Eur. J. Biochem..

[b88] Perez-Martinez L, Jaworski DM (2005). Tissue inhibitor of metalloproteinase-2 promotes neuronal differentiation by acting as an anti-mitogenic signal. J. Neurosci..

[b89] Prokhortchouk A, Hendrich B, Jorgensen H, Ruzov A, Wilm M, Georgiev G, Bird A, Prokhortchouk E (2001). The p120 catenin partner Kaiso is a DNA methylation-dependent transcriptional repressor. Genes Dev..

[b90] Rai K, Jafri IF, Chidester S, James SR, Karpf AR, Cairns BR, Jones DA (2010). Dnmt3 and G9a cooperate for tissue-specific development in zebrafish. J. Biol. Chem..

[b91] Ramocki MB, Peters SU, Tavyev YJ, Zhang F, Carvalho CM, Schaaf CP, Richman R, Fang P, Glaze DG, Lupski JR, Zoghbi HY (2009). Autism and other neuropsychiatric symptoms are prevalent in individuals with MeCP2 duplication syndrome. Ann. Neurol..

[b92] Reichwald K, Thiesen J, Wiehe T, Weitzel J, Poustka WA, Rosenthal A, Platzer M, Stratling WH, Kioschis P (2000). Comparative sequence analysis of the MECP2-locus in human and mouse reveals new transcribed regions. Mamm. Genome.

[b93] Sempere LF, Freemantle S, Pitha-Rowe I, Moss E, Dmitrovsky E, Ambros V (2004). Expression profiling of mammalian microRNAs uncovers a subset of brain-expressed microRNAs with possible roles in murine and human neuronal differentiation. Genome Biol..

[b94] Skene PJ, Illingworth RS, Webb S, Kerr AR, James KD, Turner DJ, Andrews R, Bird AP (2010). Neuronal MeCP2 is expressed at near histone-octamer levels and globally alters the chromatin state. Mol. Cell.

[b95] Szulwach KE, Li X, Smrt RD, Li Y, Luo Y, Lin L, Santistevan NJ, Li W, Zhao X, Jin P (2010). Cross talk between microRNA and epigenetic regulation in adult neurogenesis. J. Cell Biol..

[b96] Taganov KD, Boldin MP, Chang KJ, Baltimore D (2006). NF-kappaB-dependent induction of microRNA miR-146, an inhibitor targeted to signaling proteins of innate immune responses. Proc. Natl Acad. Sci. USA.

[b97] Takizawa T, Nakashima K, Namihira M, Ochiai W, Uemura A, Yanagisawa M, Fujita N, Nakao M, Taga T (2001). DNA methylation is a critical cell-intrinsic determinant of astrocyte differentiation in the fetal brain. Dev. Cell.

[b98] Tao J, Hu K, Chang Q, Wu H, Sherman NE, Martinowich K, Klose RJ, Schanen C, Jaenisch R, Wang W, Sun YE (2009). Phosphorylation of MeCP2 at Serine 80 regulates its chromatin association and neurological function. Proc. Natl Acad. Sci. USA.

[b99] Thornton P, Pinteaux E, Gibson RM, Allan SM, Rothwell NJ (2006). Interleukin-1-induced neurotoxicity is mediated by glia and requires caspase activation and free radical release. J. Neurochem..

[b100] Tsujimura K, Abematsu M, Kohyama J, Namihira M, Nakashima K (2009). Neuronal differentiation of neural precursor cells is promoted by the methyl-CpG-binding protein MeCP2. Exp. Neurol..

[b101] Turek-Plewa J, Jagodzinski PP (2005). The role of mammalian DNA methyltransferases in the regulation of gene expression. Cell. Mol. Biol. Lett..

[b102] Urdinguio RG, Fernandez AF, Lopez-Nieva P, Rossi S, Huertas D, Kulis M, Liu CG, Croce C, Calin GA, Esteller M (2010). Disrupted microRNA expression caused by Mecp2 loss in a mouse model of Rett syndrome. Epigenetics.

[b103] Vaissiere T, Sawan C, Herceg Z (2008). Epigenetic interplay between histone modifications and DNA methylation in gene silencing. Mutat. Res..

[b104] Vo N, Klein ME, Varlamova O, Keller DM, Yamamoto T, Goodman RH, Impey S (2005). A cAMP-response element binding protein-induced microRNA regulates neuronal morphogenesis. Proc. Natl Acad. Sci. USA.

[b105] Wade PA, Gegonne A, Jones PL, Ballestar E, Aubry F, Wolffe AP (1999). Mi-2 complex couples DNA methylation to chromatin remodelling and histone deacetylation. Nat. Genet..

[b106] Walsh CP, Chaillet JR, Bestor TH (1998). Transcription of IAP endogenous retroviruses is constrained by cytosine methylation. Nat. Genet..

[b107] Wang H, Chan SA, Ogier M, Hellard D, Wang Q, Smith C, Katz DM (2006). Dysregulation of brain-derived neurotrophic factor expression and neurosecretory function in Mecp2 null mice. J. Neurosci..

[b108] Watt F, Molloy PL (1988). Cytosine methylation prevents binding to DNA of a HeLa cell transcription factor required for optimal expression of the adenovirus major late promoter. Genes Dev..

[b109] Wiesel TN (1982). Postnatal development of the visual cortex and the influence of environment. Nature.

[b110] Yoshii A, Constantine-Paton M (2010). Postsynaptic BDNF-TrkB signaling in synapse maturation, plasticity, and disease. Dev. Neurobiol..

[b111] Zhang Y, Ng HH, Erdjument-Bromage H, Tempst P, Bird A, Reinberg D (1999). Analysis of the NuRD subunits reveals a histone deacetylase core complex and a connection with DNA methylation. Genes Dev..

[b112] Zhang J, Pho V, Bonasera SJ, Holtzman J, Tang AT, Hellmuth J, Tang S, Janak PH, Tecott LH, Huang EJ (2007). Essential function of HIPK2 in TGFbeta-dependent survival of midbrain dopamine neurons. Nat. Neurosci..

[b113] Zheng W, Nowakowski RS, Vaccarino FM (2004). Fibroblast growth factor 2 is required for maintaining the neural stem cell pool in the mouse brain subventricular zone. Dev. Neurosci..

[b114] Zhou Z, Hong EJ, Cohen S, Zhao WN, Ho HY, Schmidt L, Chen WG, Lin Y, Savner E, Griffith EC, Hu L, Steen JA, Weitz CJ, Greenberg ME (2006). Brain-specific phosphorylation of MeCP2 regulates activity-dependent Bdnf transcription, dendritic growth, and spine maturation. Neuron.

[b115] Zoghbi HY (2003). Postnatal neurodevelopmental disorders: meeting at the synapse?. Science.

